# The osteogenesis-promoting effects of alpha-lipoic acid against glucocorticoid-induced osteoporosis through the NOX4, NF-kappaB, JNK and PI3K/AKT pathways

**DOI:** 10.1038/s41598-017-03187-w

**Published:** 2017-06-13

**Authors:** Shi-Yu Lu, Chang-Yuan Wang, Yue Jin, Qiang Meng, Qi Liu, Zhi-hao Liu, Ke-Xin Liu, Hui-Jun Sun, Mo-Zhen Liu

**Affiliations:** 1 Department of Clinical Pharmacology, College of Pharmacy, Dalian Medical University, Dalian, China; 2 Department of Orthopaedics, First Affiliated Hospital, Dalian Medical University, Dalian, China

## Abstract

Recently, accumulating evidence has indicated that glucocorticoid-induced osteoporosis (GIOP) is closely related to oxidative stress and apoptosis. Alpha-lipoic acid (LA), a naturally endogenous anti-oxidant, possesses anti-oxidative and anti-apoptosis activities, implicating LA as a therapeutic agent for the treatment of GIOP. In this study, the osteogenesis-promoting effects of LA against GIOP were investigated and the mechanisms were further probed. Here, the results showed that LA inhibited oxidative stress, suppressed apoptosis and improved osteopenia by promoting the expression of osteogenesis markers, including ALP, COL-I, OCN, BMP-2, RUNX2 and OSX. Further study revealed that the osteogenesis-promoting effects of LA likely occur via the regulation of the NOX4, NF-kappaB, JNK and PI3K/AKT pathways. The present study indicated that LA may prevent GIOP and promote osteogenesis and might be a candidate for the treatment of GIOP.

## Introduction

Glucocorticoids (GCs) are widely used as immunosuppressive and anti-inflammatory drugs to treat various diseases, such as autoimmune disorders, inflammation and cancer. However, excessive or prolonged GC treatment can lead to an increased risk of bone fractures and osteoporosis (OP)^1, 2^. Approximately 30–50% of patients receiving prolonged GC therapy develop glucocorticoid-induced osteoporosis (GIOP)^3, 4^.

Osteoporosis is a skeletal disease that is characterized by the deterioration of the micro-architecture and low bone mass, leading to bone strength reduction and increased fracture risk^5^. The balance of bone metabolism that depends on the interactions between osteoclasts and osteoblasts is maintained by the balance between bone formation and bone resorption. Thus, bone metabolism disorders are caused by an imbalance between bone resorption and bone formation^6, 7^. Excessive or prolonged GC treatment, for instance, with dexamethasone, has been considered to play a major role in bone formation inhibition, and GIOP has been identified as the most common type of secondary osteoporosis^8^. The underlying mechanism of GIOP has not been confirmed^9^, but oxidative stress is one of the potential mechanisms in GIOP^3^. Dexamethasone (DEX) can indirectly induce oxidative stress via inhibiting antioxidant enzymes or depleting antioxidant molecules^10, 11^. DEX can also result in GIOP via inducing apoptosis^12–14^. ROS, metabolic reaction by-products that can induce oxidative stress and are pro-apoptotic, play crucial roles in the NOX4, NF-kappaB, JNK and PI3K/AKT pathways^15, 16^.

NADPH oxidase, the major source of ROS, has several isoforms: Nox1-5, Duox 1 and Duox 2. NOX4 is a constitutively active NOX enzyme that serves as an oxygen sensor and plays a major role in the generation of ROS to inhibit bone formation^17^. Therefore, antioxidants inhibiting the generation of ROS by down-regulating the NOX4 protein expression level may have an effect on bone formation promotion in GIOP.

Nuclear factor-kappaB (NF-kappaB), a multi-functional transcription factor that is activated by oxidative stress, regulates the expression of various genes involved in numerous cellular activities^18, 19^. The role of NF-kappaB signalling has not been as extensively studied in osteoblastic bone formation as it has been in osteoclastic bone resorption. A prior study demonstrated that oxidative stress induces NF-kappaB activation in MC3T3-E1 cells and OVX rats^20^; however, whether the activation of NF-kappaB is inhibited by LA in GIOP remains elusive. Mitogen-activated protein kinase (MAPK) pathways, such as c-Jun N-terminal kinases (JNK), are involved in the progression of cell apoptosis in osteoblasts^21^. However, whether the mechanism of LA on osteoblastic bone formation and apoptosis in GIOP is via the JNK signalling pathways remains unclear.

The phosphatidylinositol 3-kinase/Akt pathway is also viewed as a crucial factor for osteoblast apoptosis^22^. Notably, the inhibition of PI3K and subsequent phos-AKT appear to be the key mechanisms of apoptosis. However, whether the activation of the PI3K/AKT pathway is affected by LA in GIOP remains unknown.

Alpha-lipoic acid (LA), a powerful anti-oxidative agent, can scavenge ROS to protect some cell types against oxidative stress and apoptosis^23, 24^. In a previous study, we found that LA promoted osteoblastic formation in H_2_O_2_-treated MC3T3-E1 cells and prevented OVX-induced bone loss in rats^20^, but whether LA prevents bone loss in GIOP remains unclear. Thus, the intention of the current study was to probe the effects of LA on preventing GIOP *in vivo* and *in vitro* to clarify the potential mechanisms of LA in promoting osteoblastic formation in DEX-treated MC3T3-E1 cells and in preventing bone loss in GIOP rats. Related markers of bone formation, corresponding parameters of oxidative stress and apoptosis, as well as the NOX4, NF-kappaB, JNK or PI3K/AKT signalling pathways were probed both *in vivo* and *in vitro*. Alendronate (ALN), a standard bisphosphonate drug for GIOP treatment that may promote the bone formation of osteoblasts and inhibit the apoptosis of osteoblasts was used as the positive control agent^25–29^.

## Results

### Bone protective effects of LA *in vivo*

#### LA inhibited GIOP

**Figure 1:**
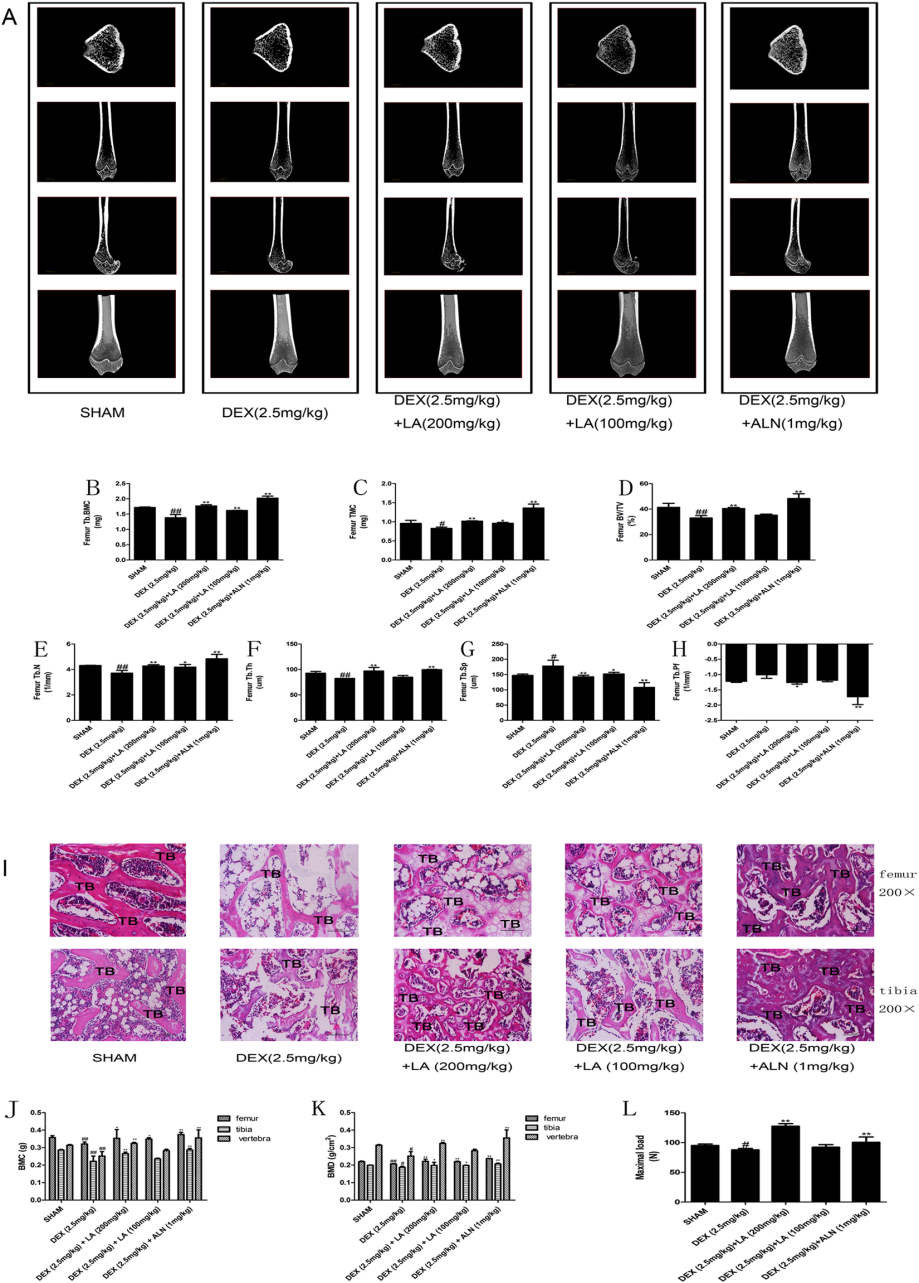
The representative micro-CT images of the femurs in each group (**A**) (n = 3 for each group). Analysis of the distal femoral trabecular bone parameters by micro-CT. Tb.BMC (**B**), TMC (**C**), BV/TV (**D**), Tb.N. (**E**), Tb.Th. (**F**), Tb.sp. (**G**), Tb.pf. (**H**) Data were the means ± SD (n = 3 for each group). ^#^ *P* < 0.05 and ^##^ *P* < 0.01, vs. the SHAM group. *P < 0.05 and **P < 0.01, vs. the DEX group. The static histomorphometric analysis of femur and tibia by hematoxylin & eosin staining (**I**) (n = 3 for each group). All images of H&E staining were at ×200 magnifications. Bars: 100 μm. TB: trabecular. BMC and BMD of femur, tibia and vertebra (n = 6 for each group). BMC were determined in each of the five groups (**J**). BMD was calculated on the basis of BMC (**K**). Evaluation of biomechanical properties on femur by using a three point blending test (**L**) (n = 3 for each group), It was calculated from load-displacement curves. ^#^ *P* < 0.05 and ^##^ *P* < 0.01, vs. the SHAM group. *P < 0.05 and **P < 0.01, vs. the DEX group.

The analysis of trabecular bone parameters using μCT indicated that DEX had significantly negative effects on the structural properties of the trabecular bone of the femur, including bone volume fraction (BV/TV), trabecular number (Tb.N.), trabecular thickness (Tb.Th.), trabecular separation (Tb.Sp.), trabecular pattern factor (Tb.pf.), as well as tissue mineral content (TMC) and trabecular bone mineral content (Tb.BMC.), compared with the sham group (Fig. 1A–H). Treatment with LA or ALN remarkably alleviated the negative structural properties of the trabecular bone compared with the DEX group.

In addition, to observe the changes in the tibia and femur trabecular regions, the histomorphology was examined using H&E staining (Fig. 1I), and a reduced thickness, less abundant and enhanced separation of the trabecular bone on the femur and tibia epiphysis were observed in GIOP rats. However, LA or ALN treatment clearly ameliorated the deleterious effects of DEX on the bone trabecular microstructure compared with the DEX group.

To further evaluate the effects of LA on GIOP, the BMC and BMD were examined using DXA. As shown in Fig. 1J,K, the BMC and BMD values for the femur, tibia, and lumbar vertebra were significantly reduced in the DEX group compared with the sham group. However, the administration of LA or ALN obviously elevated the BMC and BMD values compared with the DEX group.

The three-point bend test was used to evaluate the bone protective effect of LA on the maximum load of the femur. The results presented in Fig. 1L obviously showed that DEX exposure led to a significant reduction in the maximum load compared with the sham rats, which was clearly enhanced following LA or ALN treatment.

### Effects of LA on the antioxidant enzyme activity, oxidative injury and apoptosis in GIOP rats

**Figure 2:**
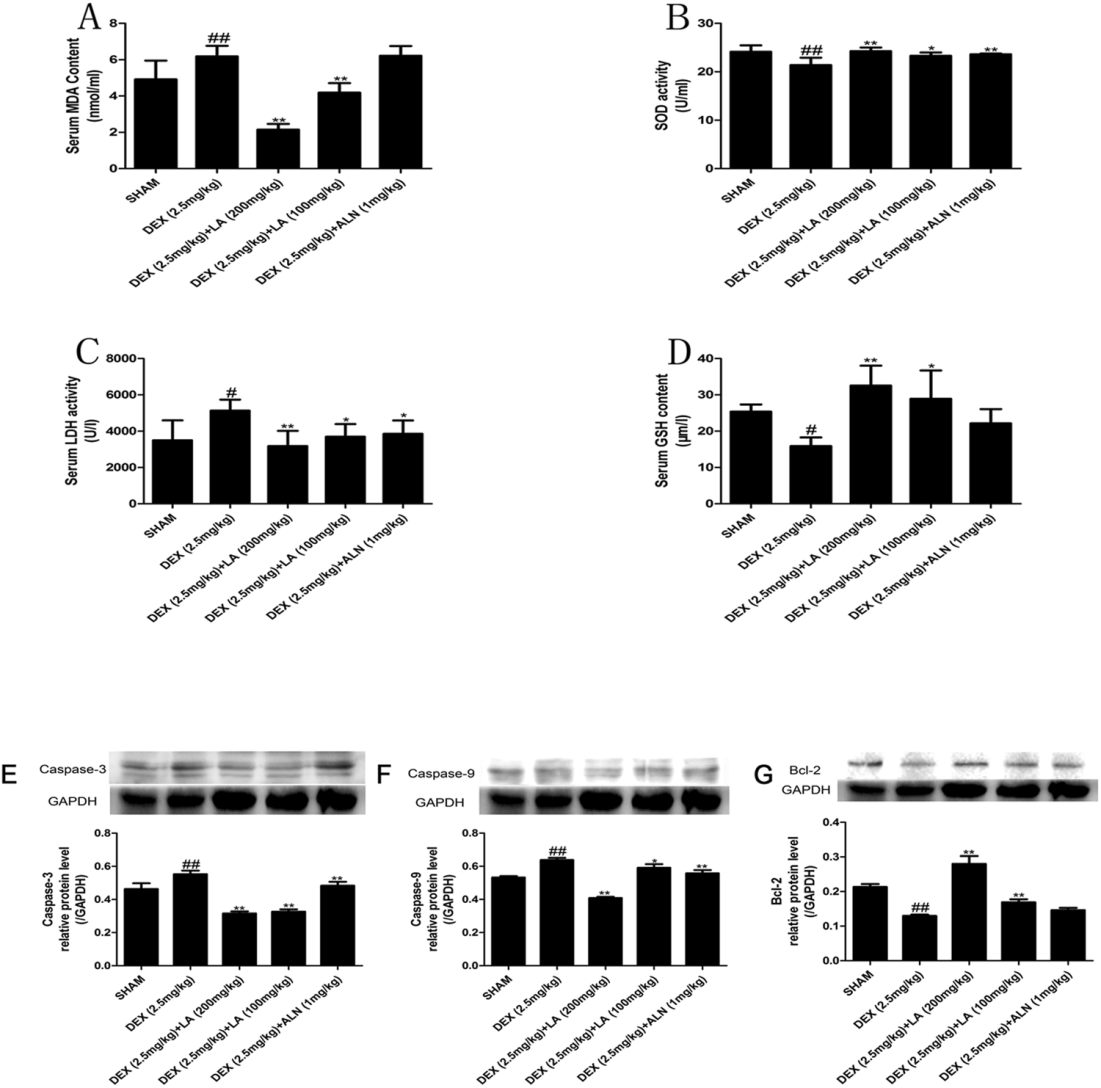
Analysis of the serum oxidant and antioxidant parameters. MDA (**A**), SOD (**B**), LDH (**C**), GSH (**D**). Data were the means ± SD (n = 6 for each group)^#^.*P* < 0.05 and ^##^ *P* < 0.01, vs. the SHAM group.*P < 0.05 and **P < 0.01, vs. the DEX group. Effect of LA on apoptosis protein (Caspase-3, Caspase-9, Bcl-2) expression on the femur. The protein expression levels of Caspase-3 (**E**), Caspase-9 (**F**), Bcl-2 (**G**) were detected by western blotting. GAPDH was used as the loading control. Data were the means ± SD (n = 6 for each group). ^##^ *P* < 0.01, vs. the SHAM group. **P < 0.01, vs. the DEX group.

Because of the instability of ROS induced by DEX *in vivo*, it is difficult to measure ROS in the serum or tissue of rats. Therefore, the anti-oxidative markers SOD and GSH and the oxidative injury markers MDA and LDH were evaluated in the serum. The results showed that the GC led to the evident inhibition of SOD and GSH and to an obvious enhancement of MDA and LDH, but the above-mentioned effects of DEX could be significantly reversed by LA. The MDA and GSH content could not be increased by ALN compared with the sham group (Fig. 2A–D).

To evaluate the effects of LA on the apoptosis induced by GIOP *in vivo*, the anti-apoptotic protein Bcl-2 and the pro-apoptotic proteins caspase-9 and caspase-3 were examined by western blotting. As shown in Fig. 2E-G﻿ and Supplemental Fig. S1-4,the expression of Bcl-2 in the DEX group was extremely decreased compared with the sham group, but the expression of Bcl-2 in the LA group was significantly increased compared with the DEX group. Moreover, the expression of caspase-9 and caspase-3 showed the opposite result. LA or ALN significantly reduced the over-expression of caspase-9 and caspase-3 induced by DEX treatment.

### LA increased the OPG/RANKL ratio and alleviated Lrp5/beta-catenin signalling pathway inhibition in GIOP rats

**Figure 3:**
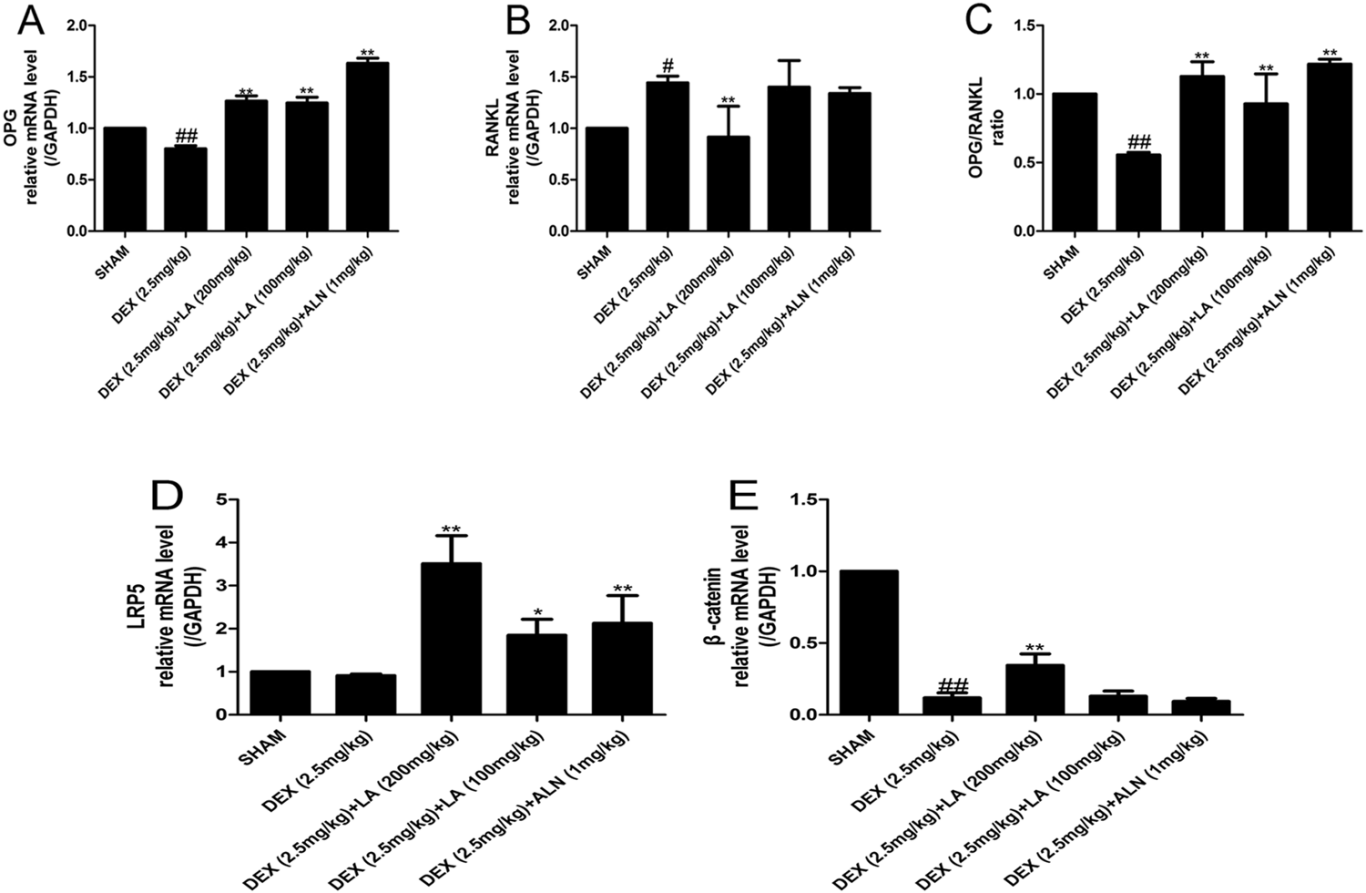
The mRNA expression levels of OPG and RANKL on the femur. The mRNA expression levels were measured by qRT-PCR for OPG (**A**), RANKL (**B**). OPG/RANKL ratio (**C**). Data were the means ± SD (n = 6 for each group). ^#^ *P* < 0.05 and ^##^ *P* < 0.01, vs. the SHAM group. *P < 0.05 and **P < 0.01, vs. the DEX group. The mRNA expression levels of the LRP5/beta-catenin pathway on femur. The mRNA expression levels were measured by qRT-PCR for LRP5 (D), beta-catenin (E). Data were the means ± SD (n = 6 for each group). ^#^ *P* < 0.05 and ^##^ *P* < 0.01, vs. the SHAM group. *P < 0.05 and **P < 0.01, vs. the DEX group.

The OPG/RANKL ratio plays a key role in regulating the process of bone remodelling^30^. As shown in Fig. 3A–C, the mRNA expression level of OPG and the ratio of OPG/RANKL in the DEX group were significantly decreased, and the mRNA expression level of RANKL was significantly increased compared with the sham group. However, the effects of DEX could be intensely reversed by LA or ALN.

Given the importance of the LRP5/beta-catenin pathway in osteoblastic formation^31^, the effects of LA on the LRP5/beta-catenin osteogenic signalling pathway were also assessed. As shown in Fig. 3D,E, the mRNA expression levels of beta-catenin in the DEX group were remarkably decreased compared with the sham group, but both LA and ALN substantially increased the mRNA expression levels of LRP5 and beta-catenin compared with the DEX group.

### LA increased the expression levels of bone formation markers in GIOP rats

**Figure 4:**
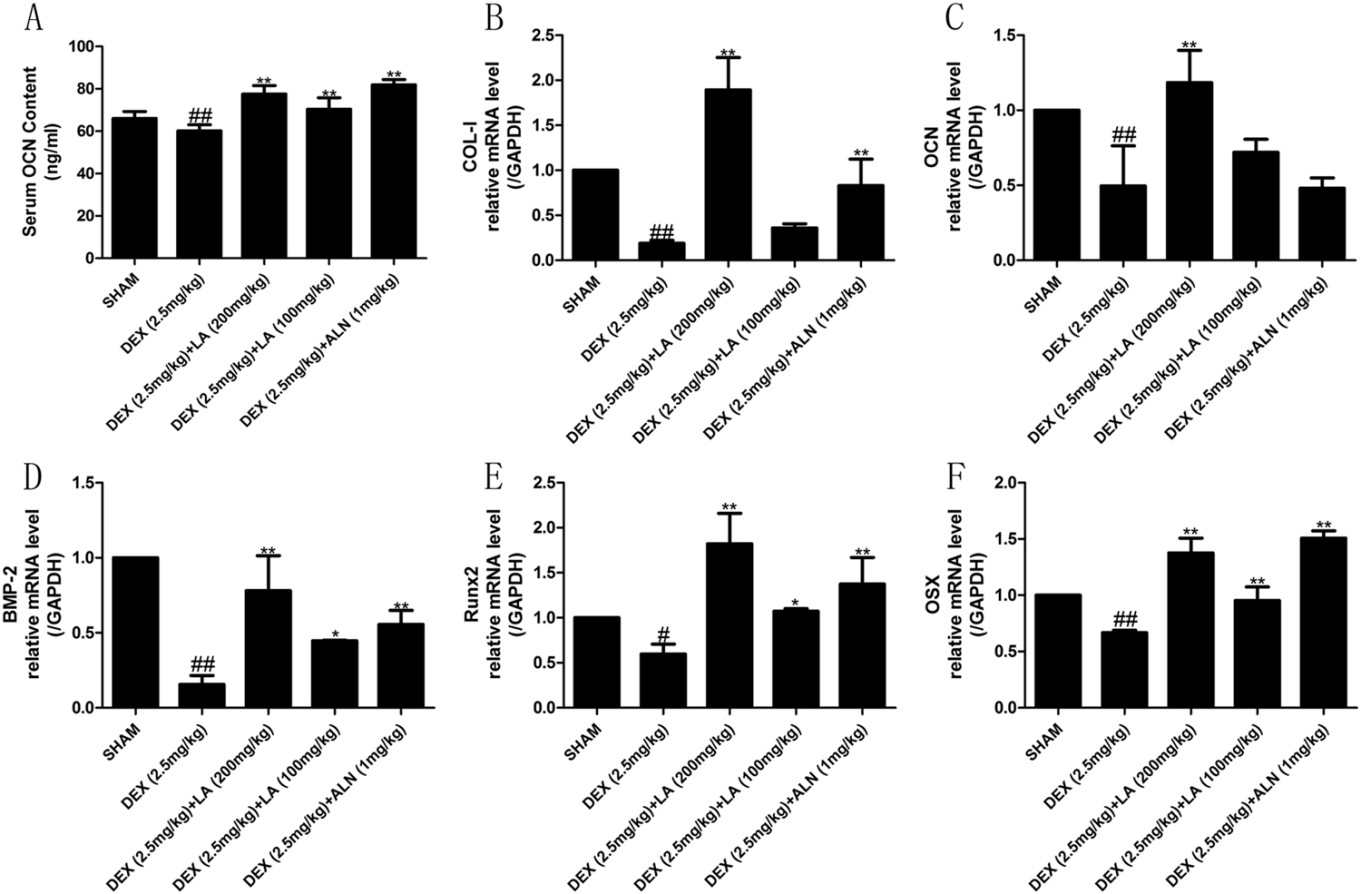
The expression of bone formation related markers. Analysis of serum OCN levels (**A**). The mRNA expression levels on femur were measured by qRT-PCR for bone COL-I (**B**), OCN (**C**), BMP-2 (**D**), RUNX2 (**E**) and OSX (**F**). Data were the means ± SD (n = 6 for each group). ^#^ *P* < 0.05 and ^##^ *P* < 0.01, vs. the SHAM group. *P < 0.05 and **P < 0.01, vs. the DEX group.

Bone formation inhibition caused by DEX is considered to play a major role in GIOP, and the levels of serum OCN are widely used as bone formation markers^32, 33^. Therefore, serum OCN was evaluated in an *in* vivo study. As shown in Fig. 4A, compared with the sham group, there was a significant reduction in the serum levels of OCN in the DEX group. However, treatment with LA or ALN improved the level of OCN compared with the DEX group.

The effects of LA on bone formation markers, including COL-I, OCN, BMP-2, RUNX2 and OSX, were evaluated. The result showed that the mRNA levels of the bone formation genes COL-I, OCN, BMP-2, RUNX2 and OSX were evidently decreased in the DEX group compared with the sham group, whereas LA and ALN obviously increased the mRNA levels of these bone formation makers in contrast to the DEX group (Fig. 4B–F).

### LA inhibited the activation of the Nox4, NF-kappaB, and JNK signalling pathways and alleviated the inhibition of the PI3K/AKT signalling pathway in GIOP rats

**Figure 5:**
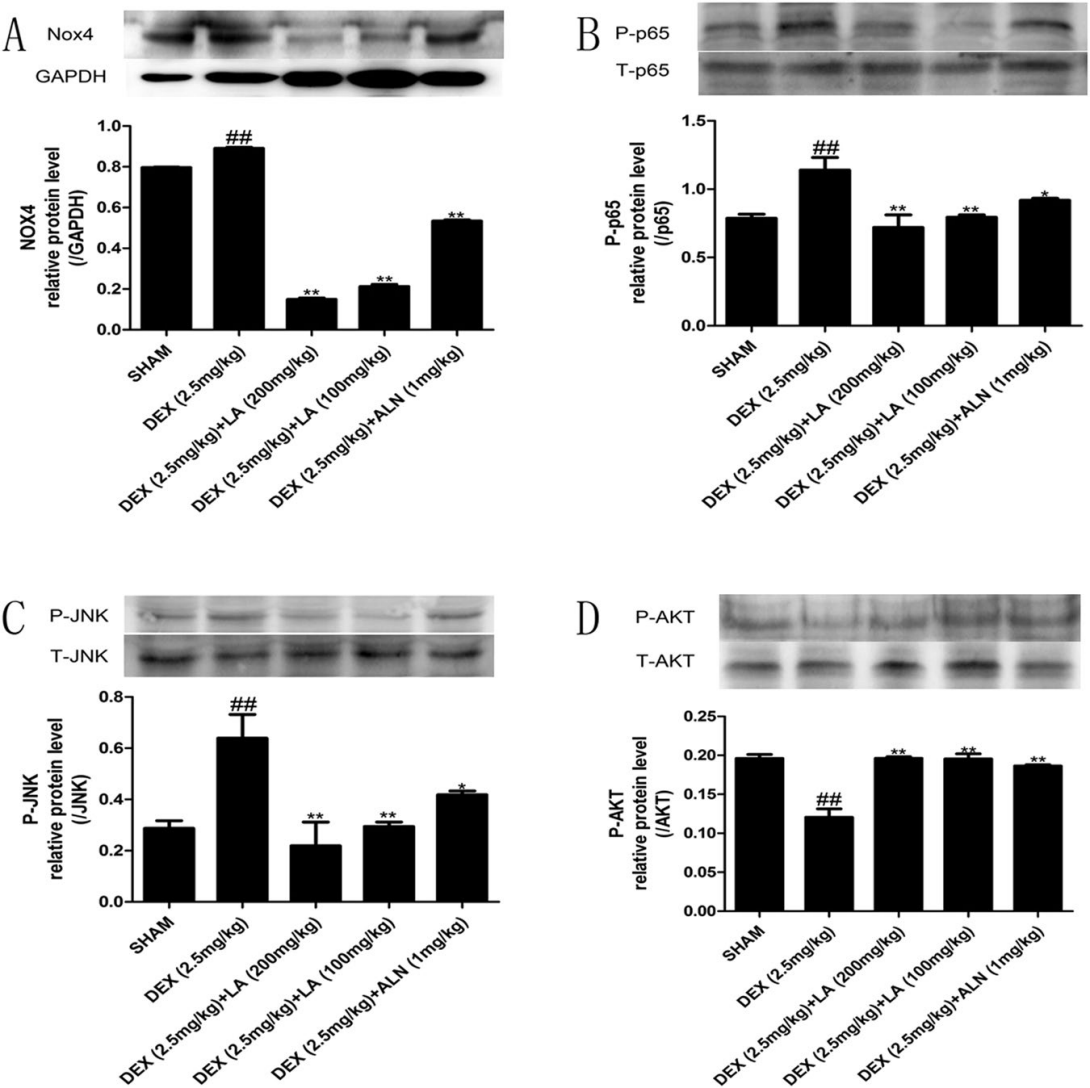
The protein expression levels of NOX4, NF-kappaB, JNK and the PI3K/AKT signalling pathway on femur. Nox4 (**A**), levels of phosphorylation of p65 (**B**), levels of phosphorylation of JNK (**C**) and levels of phosphorylation of AKT (**D**) were detected by western blotting. Data were the means ± SD (n = 6 for each group). ^#^ *p* < 0.05 and ^##^ *p* < 0.01 compared with the SHAM group. *p < 0.05 and **p < 0.01 compared with the DEX group. GAPDH was used as loading control.

NOX4 can regulate the function of osteoblasts^17^. Hence, the suppression of NOX4 activity probably antagonized GIOP *in vivo*. The western blot analysis showed that DEX up-regulated NOX4 protein expression compared with the sham group, whereas LA and ALN markedly down-regulated the NOX4 protein over-expression induced by DEX (Fig. 5A and Supplemental Fig. S5-6).

As NF-kappaB has been shown to control osteoblast function in osteoporosis^34^, the NF-kappaB activity was examined to clarify the effect of LA on the NF-kappaB signalling pathway in GIOP rats. The results indicated that DEX induced the phosphorylation of P^65^ compared with the sham group; conversely, both LA and ALN treatment obviously suppressed the phosphorylation of P^65^ protein expression compared with the DEX group (Fig. 5B﻿ and Supplemental Fig. S7-8).

The JNK signalling pathway is also involved in osteoblast formation^35^. To probe the effect of LA on the JNK signalling pathway, the phosphorylation level of JNK was examined by western blotting. DEX significantly elevated the p-JNK level in contrast to the sham group. However, LA and ALN remarkably suppressed the p-JNK level in contrast to the DEX group (Fig. 5C and Supplemental Fig. S9-10).

It has been reported that PI3K/AKT plays a crucial role in bone formation^36, 37^. Thus, the phosphorylation status of Akt was assessed. The DEX group showed a significant decrease in the p-AKT level compared with the sham group. However, LA and ALN treatment showed a significant increase in the p-AKT level compared with the DEX group (Fig. 5D and Supplemental Fig. S11-12).

### Osteoblastic-preventative effects of LA *in vitro*

#### LA protected MC3T3-E1 cells against DEX-induced oxidative injury and apoptosis

**Figure 6:**
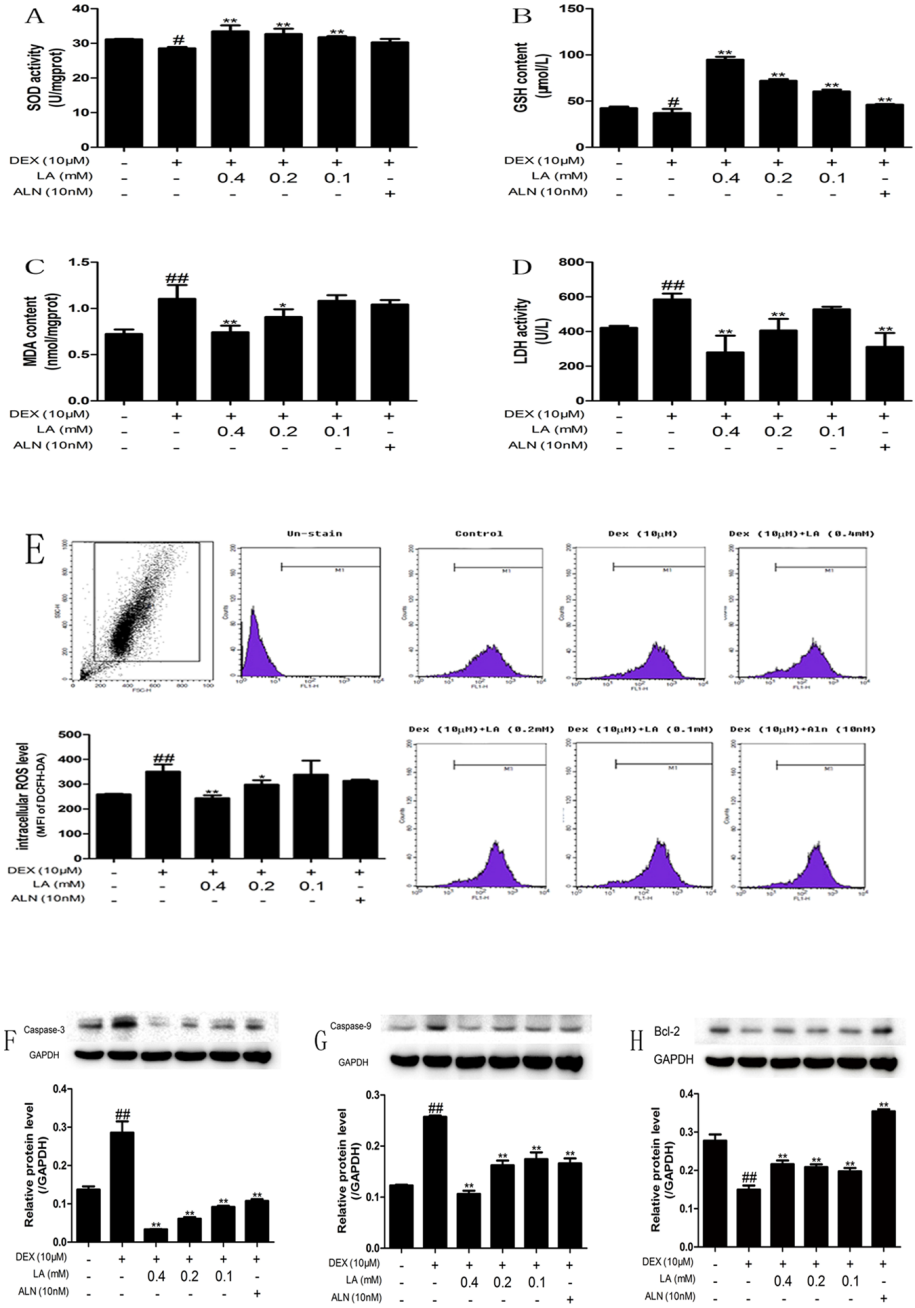
Effects of LA on biochemical parameters of oxidative stress in MC3T3-E1 cell. Cells were treated with LA or ALN while being treated with DEX. SOD (**A**), GSH (**B**), MDA (**C**) and LDH (**D**) were detected by assay kit. LA scavenged the excessive generation of intracellular ROS (**E**) stimulated by DEX. All experiments were performed three times with identical results. ^#^ *p* < 0.05 and ^##^ *p* < 0.01 compared with the control group. *p < 0.05, **p < 0.01 compared with the DEX-alone group. The expression levels of apoptosis proteins (Caspase-3, Caspase-9, Bcl-2) in MC3T3-E1 cells. Caspase-3 (**E**), Caspase-9 (**F**), Bcl-2 (**G**) were detected by western blotting. GAPDH was used as the loading control. All experiments were performed three times with identical results. ^#^ *p* < 0.05 and ^##^ *p* < 0.01 compared with the control group. *p < 0.05, **p < 0.01 compared with the DEX-alone group.

The effects of LA on oxidative stress markers, including the biochemical parameters SOD, GSH, MDA and LDH, were examined to evaluate whether LA protects MC3T3-E1 cells from oxidative injury induced by DEX. The results showed that DEX significantly decreased the SOD and GSH levels compared with the control group. By contrast, LA remarkably increased the level of SOD and GSH compared with the DEX group (Fig. 6A,B). On the other hand, the increased level of MDA and LDH induced by DEX was obviously decreased by LA (Fig. 6C,D).

To assess whether LA affects ROS generation, the intracellular ROS level was measured. As shown in Fig. 6E, DEX treatment alone remarkably increased ROS generation compared with the control group. Compared with DEX treatment alone, LA intensely attenuated DEX-induced intracellular ROS over-generation.

The levels of the apoptosis-related proteins Bcl-2, caspase-9, and caspase-3 were also estimated by western blotting to determine whether LA had protective effects against DEX-induced apoptosis in MC3T3-E1 cells. The treatment of MC3T3-E1 cells with DEX markedly increased the protein level of caspase-9 and caspase-3 compared with the control group, whereas cells exposed to LA or ALN had decreased protein levels of caspase-9 and caspase-3 compared with the DEX-alone group (Fig. 6F,G and Supplemental Fig. S13,14,16). In addition, DEX obviously suppressed the expression of the anti-apoptosis protein Bcl-2 compared with the control group. However, the LA (with DEX co-treatment) and ALN (with DEX co-treatment) groups showed significantly increased expression of Bcl-2 compared with the DEX-alone group (Fig. 6H and Supplemental Fig. S15-16).

### LA increased the OPG/RANKL ratio and activated the Lrp5/beta-catenin signalling pathway in DEX-exposed MC3T3-E1 cells

**Figure 7:**
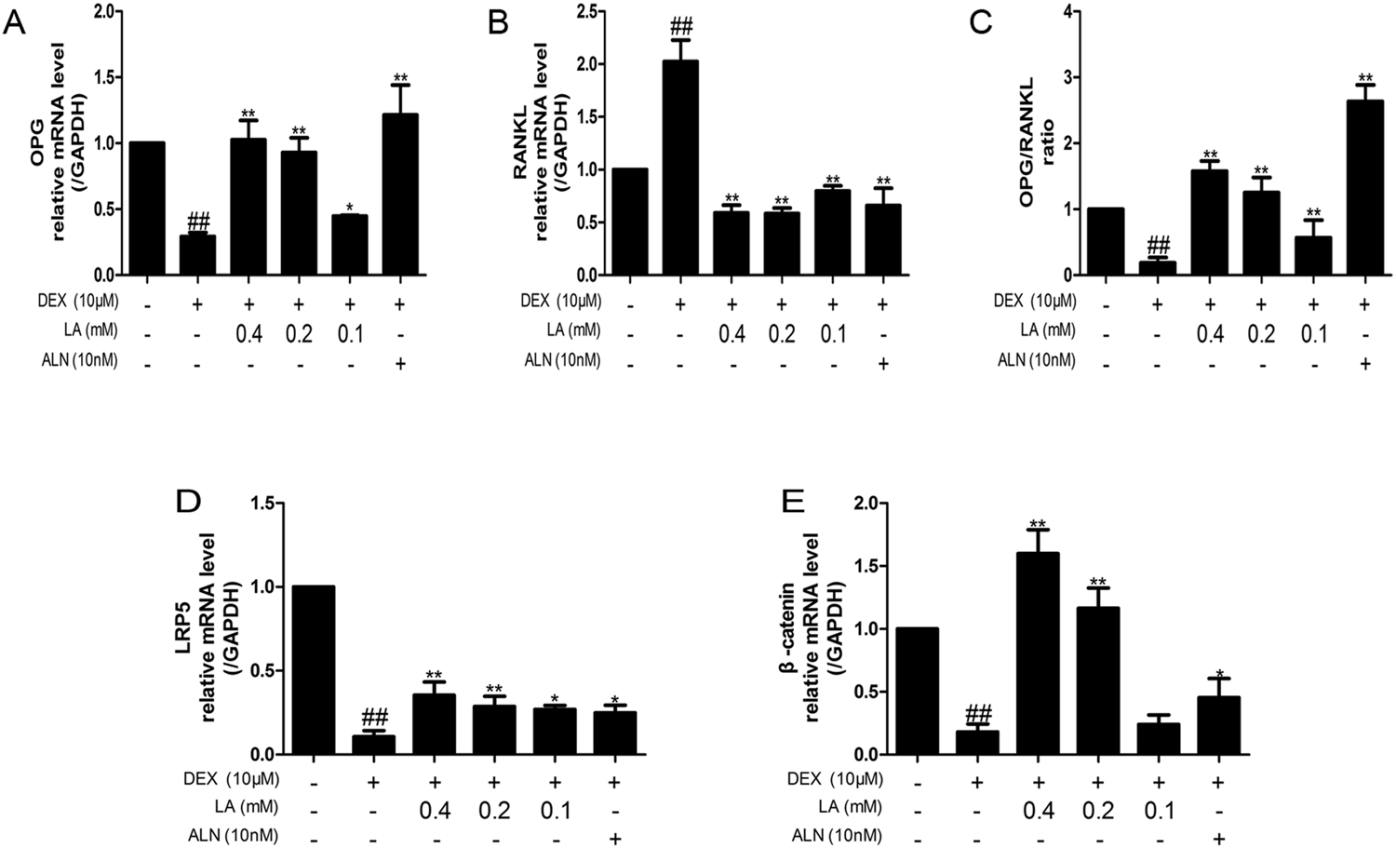
The mRNA expression levels of OPG and RANKL in MC3T3-E1 cells. OPG (**A**), RANKL (**B**) were detected by qRT-PCR. OPG/RANKL ratio (**C**). All experiments were performed three times. ^#^ *p* < 0.05 and ^##^ *p* < 0.01 compared with the control group. *p < 0.05 and **p < 0.01 compared with the DEX-alone group. The mRNA expression levels of LRP5 and beta-catenin in MC3T3-E1 cells. LRP5 (**D**) and beta-catenin (**E**) were detected by qRT-PCR. GAPDH was used as the loading control. Data were expressed as mean ± SD of three independent experiments. ^#^ *p* < 0.05 and ^##^ *p* < 0.01 compared with the control group. *p < 0.05 and **p < 0.01 compared with the DEX-alone group.

The OPG/RANKL ratio is key to the coupling of bone resorption and bone formation and has been adopted to assess bone remodelling^30^. DEX alone markedly decreased the mRNA level of OPG and increased the mRNA level of RANKL. However, the LA (with DEX co-treatment) and ALN (with DEX co-treatment) groups showed an enhancement in the mRNA level of OPG (Fig. 7A) and an inhibition in the mRNA level of RANKL (Fig. 7B). Therefore, the decreased ratio of OPG/RANKL induced by DEX was markedly increased by LA (Fig. 7C).

The mRNA levels of the LRP5/beta-catenin pathway were also detected to evaluate whether it is involved in the effects of LA on DEX-exposed MC3T3-E1 cells. As shown in Fig. 7D,E, compared with the control group, DEX significantly down-regulated the mRNA expression level of LRP5 and beta-catenin, whereas LA and ALN obviously up-regulated the mRNA expression level of LRP5 and beta-catenin compared with the DEX-alone group.

### LA enhanced the expression of osteoblastic formation markers in DEX-treated MC3T3-E1 cells

**Figure 8:**
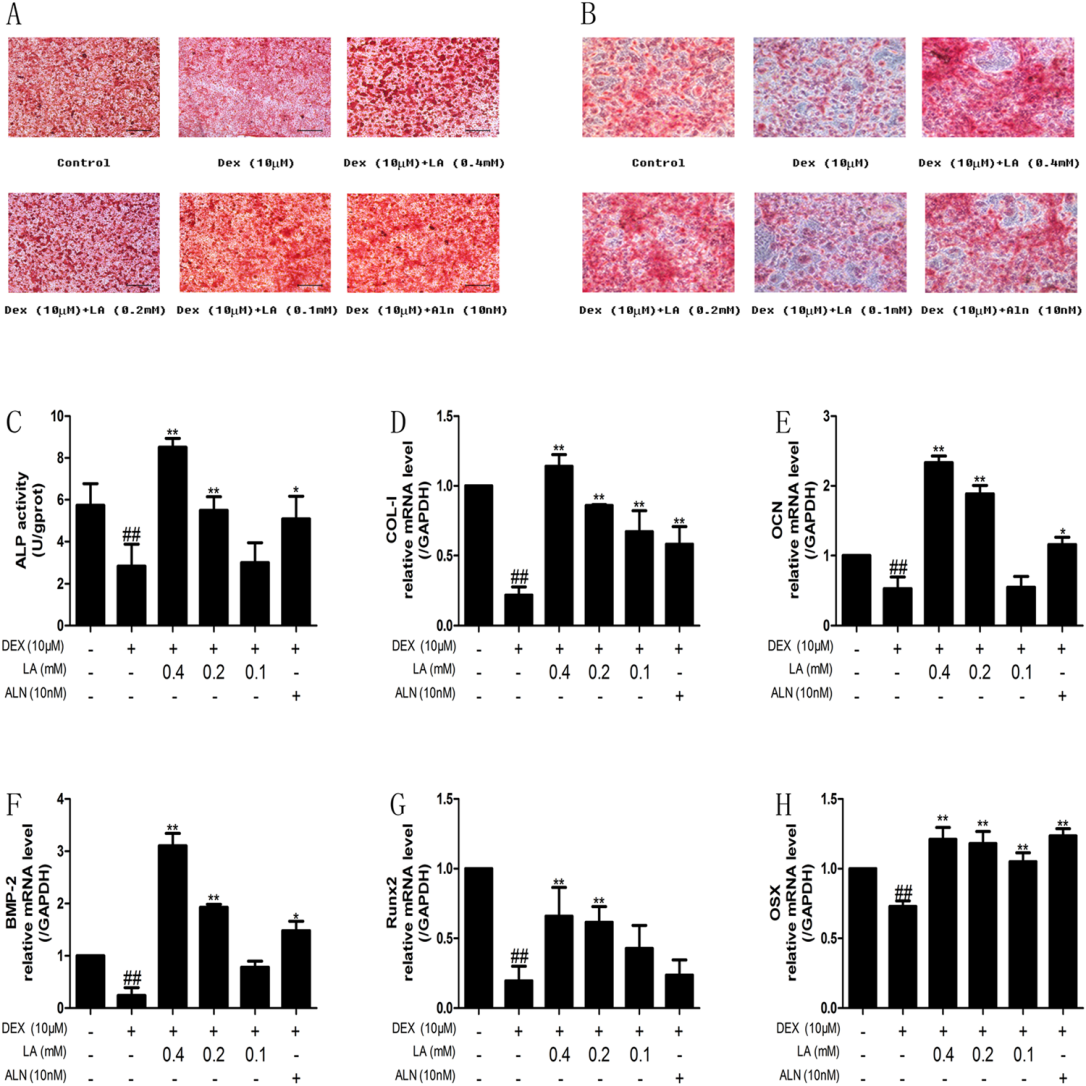
The effects of LA on bone formation markers in MC3T3-E1 cells. ALP staining (**A**), mineralization (**B**), ALP activity (**C**) and COL-I (**D**), OCN (**E**), BMP-2 (**F**), Runx2 (**G**), Osx (**H**) mRNA expression levels were detected in MC3T3-E1 cells. All experiments were performed three times. All images of ALP staining were at ×200 magnifications. Bars: 100 μm. ^#^ *p* < 0.05 and ^##^ *p* < 0.01 compared with the control group. *p < 0.05 and **p < 0.01 compared with the DEX-alone group.

To determine the effects of LA on the expression of osteoblastic formation markers, the staining and activity of ALP and the mRNA levels of COL-I, OCN, BMP-2, RUNX2 and OSX were assessed. As shown in Fig. 8A, the DEX group showed a less deeply stained region compared with the control group, and both LA and ALN could significantly increase the deeply stained region compared with the DEX-alone group. In addition, to evaluate whether LA treatment promoted osteoblastic formation on mineralized nodules in DEX-exposed MC3T3-E1 cells, mineralized nodules were stained using Alizarin red (Fig. 8B). Consistent with the results of the ALP staining, after 21 days, DEX resulted in less deeply stained regions compared with the control group, and LA or ALN led to much more deeply stained regions compared with the DEX-alone group. Cells treated with DEX exhibited decreased ALP activity and decreased mRNA expression of COL-I, OCN, BMP-2, RUNX2 and OSX compared with the control group. However, the expression of these genes intensely increased in the LA (with DEX co-treatment) and ALN (with DEX co-treatment) groups compared with the DEX-alone group (Fig. 8C–H).

### LA inhibited the NOX4, NF-kappaB, and JNK signalling pathways and activated the PI3K/AKT signalling pathway in DEX-exposed MC3T3-E1 cells

**Figure 9:**
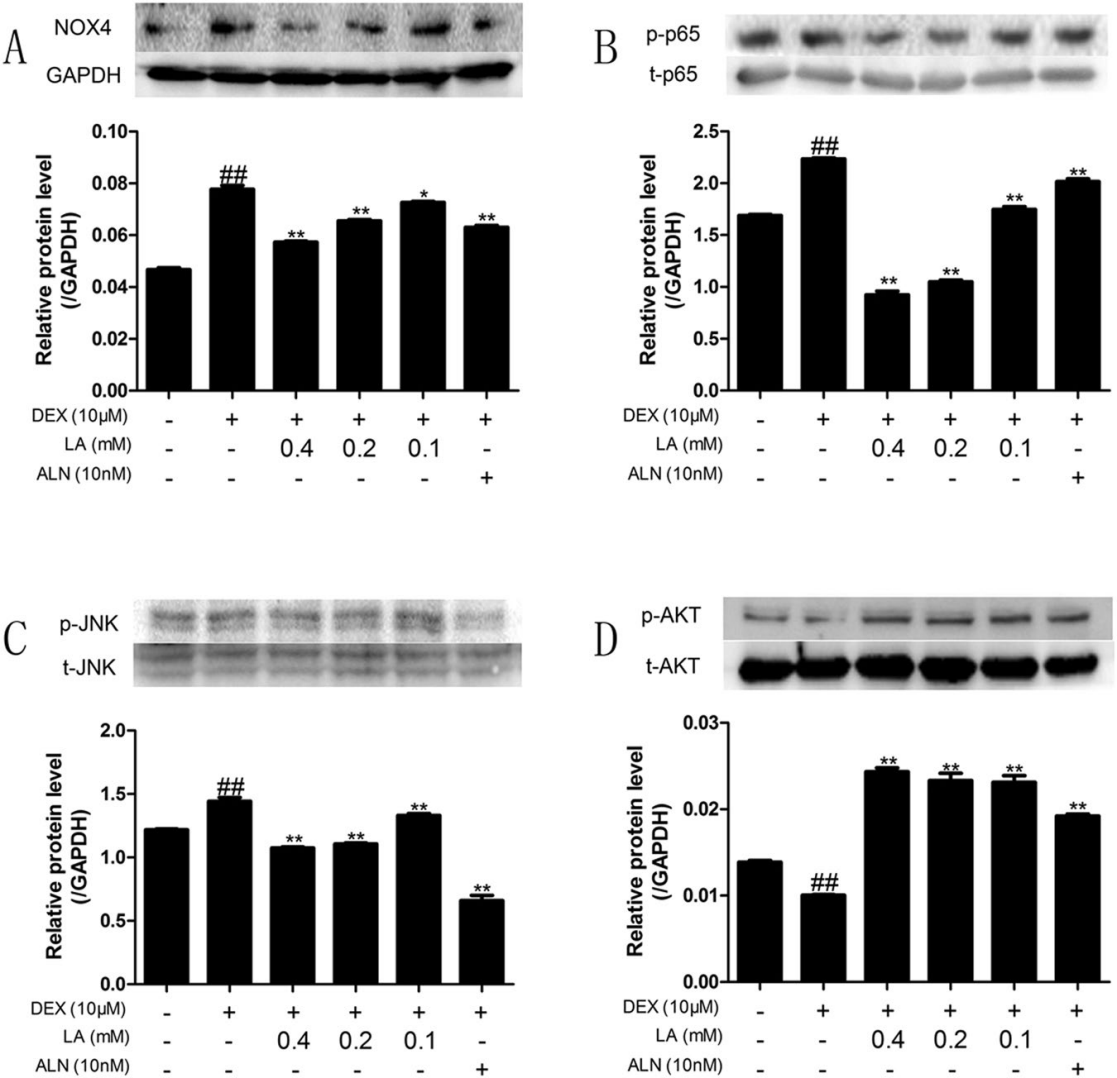
The expression levels of NOX4, NF-kappaB, JNK and the PI3K/AKT signalling pathway in MC3T3-E1 cells. NOX4 (**A**) and levels of phosphorylation of p65 (**B**) and levels of phosphorylation of JNK (**C**) and levels of phosphorylation of AKT (**D**) were detected by western blotting. GAPDH was used as loading control. Data were expressed as mean ± SD of three independent experiments. ^#^ *p* < 0.05 and ^##^ *p* < 0.01 compared with the control group. *p < 0.05 and **p < 0.01 compared with the DEX-alone group.

To investigate the effects of LA on inhibiting the NOX4, NF-kappaB and JNK signalling pathways, as well as activating the PI3K/Akt pathway, the levels of NOX4 protein expression, p65 phosphorylation, JNK phosphorylation and Akt phosphorylation were determined by western blotting (Fig. 9A–D and Supplemental Fig. S17-24). In brief, LA and ALN significantly decreased the protein expression of NOX4, P-P65, and P-JNK, and significantly increased p-Akt protein expression, compared with the DEX-alone group.

### LA enhanced osteoblastic formation by inhibiting the NOX4, NF-kappaB, and JNK pathways and activating the PI3K/AKT pathway in DEX-exposed MC3T3-E1 cells

**Figure 10:**
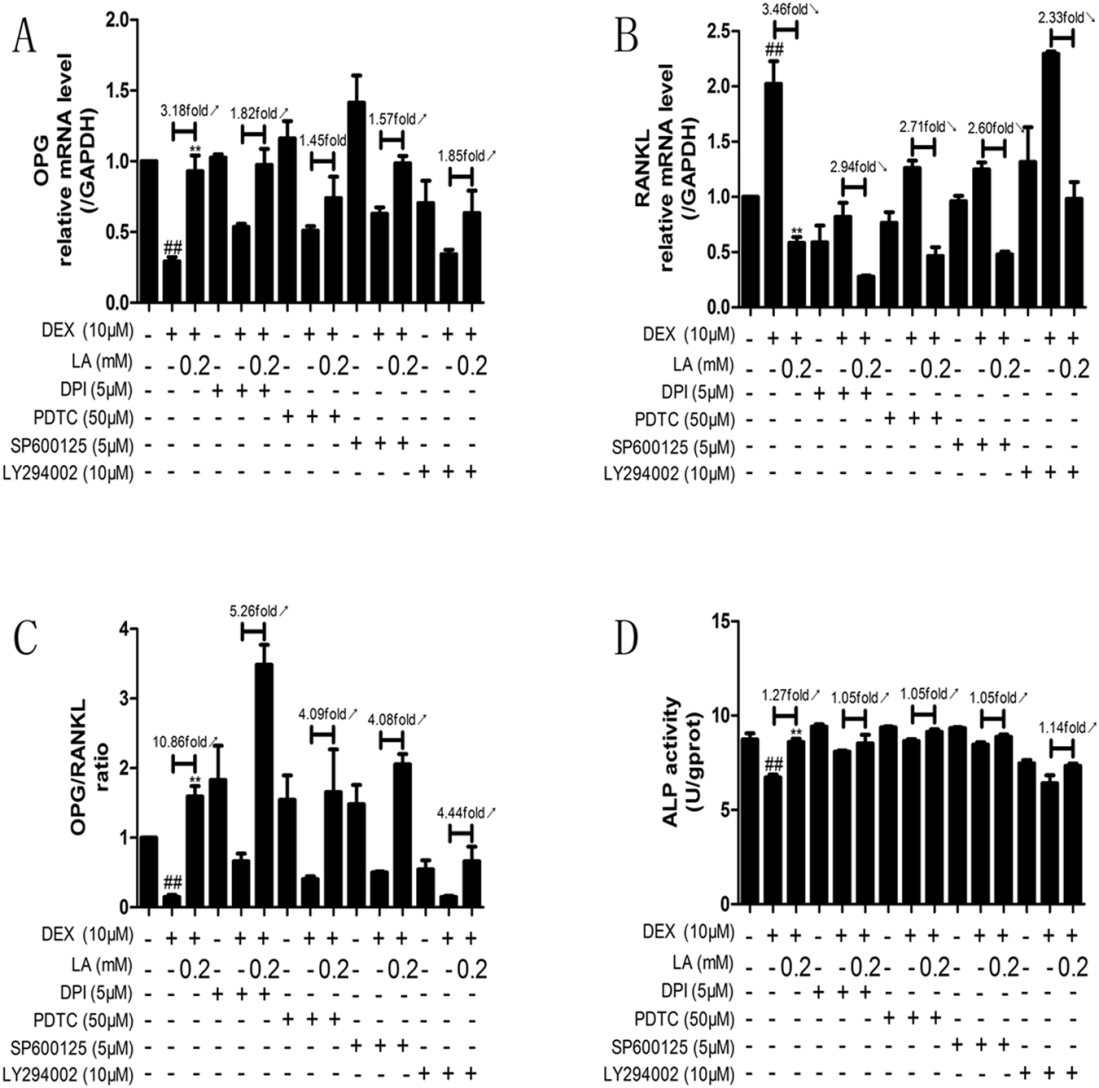
The effects which LA regulated the level of ALP activity and the ratio of OPG/ RANKL were inhibited by DPI, PDTC, LY294002 or SP600125 in DEX-treated MC3T3-E1 cells. The expression of OPG (**A**) and RANKL (**B**) mRNA was examined by qRT-PCR. OPG/RANKL ratio (**C**). ALP activity (**D**) was examined by the ALP activity assay kit. Data were expressed as mean ± SD of three independent experiments. ^#^ *p* < 0.05 and ^##^ *p* < 0.01 compared with the control group. *p < 0.05 and **p < 0.01 compared with the DEX-alone group.

To clarify whether the effects of LA on bone formation (mostly ALP activity and OPG/RANKL ratio) are through inhibiting the NOX4, NF-kappaB, and JNK pathways and activating the PI3K/AKT pathway, DPI (NOX4 inhibitor), PDTC (NF-kappaB inhibitor), SP600123 (JNK inhibitor) and LY294002 (PI3K/AKT inhibitor) were used. As shown in Fig. 10A,C,D, in normal MC3T3-E1 cells, the OPG mRNA level, the OPG/RANKL ratio and the ALP activity were decreased in the DEX-alone group compared with the control group. LA caused a 3.18-fold, 10.86-fold and 1.27-fold elevation, respectively, compared with the DEX-alone group. However, in MC3T3-E1 cells treated with DPI, LA elevated the OPG mRNA level, the OPG/RANKL ratio and the ALP activity by 1.82-fold, 5.28-fold and 1.05-fold, respectively, compared with the DEX group. In MC3T3-E1 cells treated with PDTC, LA elevated the OPG mRNA level, OPG/RANKL ratio and the ALP activity by 1.45-fold, 4.09-fold and 1.05-fold, respectively, compared with the DEX group. In MC3T3-E1 cells treated with SP600125, LA elevated the OPG mRNA level, OPG/RANKL ratio and the ALP activity by 1.57-fold, 4.08-fold and 1.05-fold, respectively, compared with the DEX group. In MC3T3-E1 cells treated with LY294002, LA elevated the OPG mRNA level, OPG/RANKL ratio and the ALP activity by 1.85-fold, 4.44-fold and 1.14-fold, respectively, compared with the DEX group. Additionally, as shown in Fig. 10B, in normal MC3T3-E1 cells, the mRNA of RANKL was increased in the DEX-alone group compared with the control group, and LA treatment resulted in 3.46-fold suppression compared with the DEX-alone group. However, in MC3T3-E1 cells treated with DPI, LA suppressed RANKL mRNA expression only by 2.94-fold compared with the DEX group. Moreover, in MC3T3-E1 cells treated with PDTC, LA suppressed RANKL mRNA expression by only 2.71-fold compared with the DEX group. In MC3T3-E1 cells treated with SP600125, LA suppressed RANKL mRNA expression by only 2.60-fold compared with the DEX group. In MC3T3-E1 cells treated with LY294002, LA suppressed the RANKL mRNA by only 2.33-fold compared with the DEX group. Collectively, these results suggested that LA suppressed RANKL over-expression and enhanced the decreased OPG expression, OPG/RANKL ratio and ALP activity induced by DEX partly by inhibiting the NOX4, NF-kappaB, and JNK pathways and activating the PI3K/AKT pathway.

## Discussion

Recently, GC administration has been considered to be the important casual matter of secondary osteoporosis^8^. Therefore, it is necessary to seek a proper method of therapy for GIOP with a lower cost and minor side effects. Laboratory rats are often used for establishing credible animal models of GC-induced osteoporosis and osteopenia^38^. In the present study, dexamethasone (DEX 2.5 mg/kg, twice a week for 9 weeks) was given to 3.5-month-old female SD rats by intramuscular injection according to a method reported previously^39^. The results of the serum OCN, BMC and BMD testing suggested that GIOP was successfully induced. MC3T3-E1 cells were cultured to study the effects of LA on bone formation in GIOP *in vitro*. At present, there are few studies that reference the effects of LA on GIOP rats or on DEX-exposed MC3T3-E1 cells.

BMC and BMD, crucial parameters reflecting bone metabolism, are widely considered predictive of fracture risk and are used to assess changes of bone mass and used to diagnose osteoporosis (OP)^40, 41^. The present study showed that LA obviously attenuated the levels of GC-induced BMC and BMD.

Osteoporosis is the result of osteopenia, and osteopenia in GIOP results from trabecular osteopenia. Hence, the micro-architecture of the trabecular bone can predict GC-induced osteopenia and the deterioration of bone quality^42^. In our *in vivo* study, LA significantly restored the trabecular connectivity by increasing Tb.N and Tb.Th., reducing Tb.sp and remarkably inhibiting the GC-induced decrease in TMC, Tb.BMC, and BV/TV. In general, LA obviously alleviated the trabecular osteopenia of GIOP rats. The obtained histomorphometric images suggested that the structural changes of trabecular bone in GIOP rats (thinning and deteriorated trabecular bone) were significantly improved by LA. Collectively, the results indicated that LA evidently improved osteopenia in GIOP rats.

The femur, the most sensitive part to atypical fractures in approximately 40% of European women treated with GC^43, 44^, was chosen to evaluate the effects of LA on GIOP. Obviously reduced bone mechanical strength and significantly increased trabecular bone damage were observed in GIOP rats. LA markedly reversed the decrease in GC-induced bone mechanical strength and reduced the risk of fractures in this study.

Oxidative stress modulates the osteoblastic differentiation of bone cells, and ROS can increase osteoblast apoptosis and can inhibit bone formation^45^. To probe the potential mechanisms of the bone formation protective effects of LA, the levels of SOD, GSH, MDA, LDH and ROS were examined *in vivo* and *in vitro*. The results suggested that LA intensely suppressed oxidative injury in DEX-exposed MC3T3-E1 cells and GIOP rats by promoting anti-oxidative defence enzymes and inhibiting oxidative stress. The anti-apoptosis effects of LA in DEX-exposed MC3T3-E1 cells and GIOP rats was further shown by the promoting effects of LA on bone formation.

The imbalance of bone metabolism leads to osteopenia, including bone formation inhibition and bone resorption promotion. The OPG/RANKL/RANK signalling pathway plays a crucial role in regulating the bone remodelling process^30^. OPG and RANKL are secreted by osteoblasts^5^. RANKL binds to RANK on osteoclasts to stimulate differentiation. OPG, a decoy receptor for RANKL, can also bind to RANKL to block this process and to control the remodelling process^46^. Hence, the OPG/RANKL ratio is key to the coupling of bone resorption and bone formation and is often used to assess bone remodelling^47^. Here, our findings suggested that the OPG/RANKL ratio could be significantly increased by LA both *in vivo* and *in vitro*. Hence, LA could possibly promote bone formation and inhibit bone resorption.

LRP5, an important coreceptor of the Wnt signalling pathway, plays a crucial role in skeletal development. Beta-catenin, a crucial downstream mediator of the signal derived from upstream LRP5^48^, can promote osteoblast differentiation and survival^49^. The Wnt signalling pathway is essential in bone maintenance and bone formation^31^. Hence, the LRP5/beta-catenin osteogenic signalling pathway was also inspected *in vivo* and *in vitro*. The results showed that the decreased mRNA level expression of LRP5 and beta-catenin induced by DEX was significantly increased by LA both *in vivo* and *in vitro*, implying that DEX-induced bone formation inhibition was alleviated by LA treatment.

Additionally, bone formation inhibition by DEX has been considered to play a major role in GIOP^9^. The serum level of OCN is widely used as a bone formation marker. Our results in the present study demonstrated that LA obviously elevated the serum OCN level in GIOP rats, implying a role of LA as a bone formation promoter.

Bone-forming osteoblasts emanate from bone mesenchymal stem cells (MSCs), and the differentiation and maturation of pre-osteoblasts (for instance, MC3T3E-1) from MSCs play a role in the re-building of resorbed bone by creating a mineralized matrix^33, 50^. To further probe the bone formation activity, the osteogenic markers in bone-forming osteoblasts were observed, including the activity of the ALP enzyme, the capacity of mineralization *in vitro*, and the mRNA levels of COL-I, OCN, BMP-2, RUNX2, and OSX *in vivo* and *in vitro* ^32, 33^. The results indicated the mRNA expression level of COL-I, OCN, BMP-2, RUNX2, and OSX was significantly increased *in vivo* and *in vitro*, and it is likely that DEX-induced bone formation inhibition could be alleviated by LA treatment.

NOX4, the major source of ROS, can regulate the function of osteoblasts^17^. Hence, the suppression of NOX4 activity could probably inhibit GIOP *in vivo* and promote bone formation *in vitro*. In the present study, LA suppressed the over-expression of Nox4, and an inhibitor of Nox4, DPI, reduced the extent to which LA promoted bone formation in DEX-exposed MC3T3-E1 cells. Our results suggested that LA inhibited GIOP *in vivo* and prevented the GC-induced inhibition of bone formation *in vitro* (mostly through ALP activity and OPG/RANKL ratio in this study) partly via suppressing the protein level expression of NOX4.

NF-kappaB has been shown to control the function of osteoblasts in osteoporosis^34^. The phosphorylation of p65 was crucial for the transactivation of p65^51^. In our study, we found that GC-induced NF-kappaB activation occurred through the increased phosphorylation of p65. LA inhibited GC-induced NF-kappaB activation by suppressing the phosphorylation of p65. PDTC, an inhibitor of the NF-kappaB signalling pathway, partly inhibited the LA-induced bone formation promoting effects in DEX-exposed MC3T3-E1 cells, suggesting that LA prevents GIOP *in vivo* and promotes bone formation (mostly through ALP activity and OPG/RANKL ratio in this study) *in vitro* partly via suppressing the activation of the NF-kappaB signalling pathway.

JNK is also involved in osteoblast formation^35^. Our results indicated that LA attenuated the GC-induced activation of p-JNK *in vivo* and *in vitro*, and pretreatment with an inhibitor of JNK (SP600125) inhibited the effect of LA attenuation in DEX-exposed MC3T3-E1, suggesting that the JNK signalling pathway plays an important role in bone formation in DEX-exposed MC3T3-E1 cells. Moreover, an inhibitor of the JNK signalling pathway (SP600125) significantly reduced the LA-dependent enhancement of ALP activity and the OPG/RANKL ratio *in vitro*, implying that LA prevented GIOP *in vivo* and promoted bone formation (mostly through the ALP activity and OPG/RANKL ratio in this study) *in vitro* possibly by suppressing the activation of the JNK signalling pathway.

It has been reported that the PI3K/AKT pathway plays a crucial role in bone formation^36, 37^. Here, it was shown that LA markedly reversed the GC-induced inhibition of p-AKT *in vivo* and *in vitro*, and that pretreatment with the inhibitor of PI3K/AKT signalling pathway (LY294002) antagonized the promotion of LA-induced bone formation in DEX-exposed MC3T3-E1 cells, revealing that the PI3K/AKT signalling pathway played a crucial role in bone formation in DEX-exposed MC3T3-E1 cells. Additionally, the inhibitor of the PI3K/AKT signalling pathway reduced the LA-dependent enhancement of ALP activity and the OPG/RANKL ratio *in vitro*, suggesting that LA prevented GIOP *in vivo* and promoted bone formation (mostly through the ALP activity and the OPG/RANKL ratio in this study) *in vitro* partly by activating the PI3K/AKT signalling pathway.

In summary, LA may prevent GIOP and promote bone formation by antagonizing oxidative stress and suppressing apoptosis. The mechanisms of LA for bone formation promotion (mostly the ALP activity and OPG/RANKL ratio in this study) possibly proceed by inhibiting the expression of the NOX4 protein and the NF-kappaB and JNK pathways and activating the PI3K/AKT signalling pathway. The present study suggests that LA treatment may be a reasonable therapeutic strategy for GIOP and would provide certain underlying benefits for GIOP patients.

## Materials and Methods

### Reagents

Dexamethasone (DEX) and DMSO were purchased from Sigma Chemical Co. (St. Louis, MO). Alpha-lipoic acid (LA) (≥99.5% purity) was purchased from Fushilai Medicine & Chemical Co., Ltd. (Changshu, China). Alendronate (ALN) was purchased from J&K Scientific Ltd. (Beijing, China). Beta-glycerophosphate and ascorbic acid (VC) were purchased from Solarbio Science & Technology Co., Ltd. (Beijing, China). Dulbecco’s modified Eagle’s medium (DMEM) was purchased from Gibco-BRL Company (Gaithersburg, MD). Foetal bovine serum (FBS) was purchased from Haoyang Biologicals Technology Co., Ltd. (Tianjin, China). The DCFH-DA fluorescent probe, and inhibitors (DPI and PDTC) were purchased from Beyotime Institute of Biotechnology (Haimen, China). Antibodies specific for Bcl-2 (Catalog No. 12789-1-AP), caspase-9 (Catalog No. 10380-1-AP), caspase-3 (Catalog No. 19677-1-AP), NOX4 (Catalog No. 14347-1-AP), JNK (Catalog No. 51151-1-AP), and AKT (Catalog No. 10176-2-AP) were purchased from Proteintech Group (Wuhan, China). Antibodies specific for p65 (Catalog No. BS3648), phospho-p65 (Catalog No. BS4737), phospho-JNK (Catalog No. BS4763), phospho-AKT (Catalog No. BS4647), and GAPDH (Catalog No. AP0063) were purchased from Bioworld Technology (Nanjing, China). The antibody specific for goat anti-rabbit HRP-IgG (Catalog No. BS-0294R-HRP) was purchased from Biosynthesis Biotechnology Co., Ltd. (Beijing, China). The ECL Western Blotting Detection Substrate, the All-in-One cDNA Synthesis SuperMix and 2× SYBR Green qPCR Master Mix (Low ROX) were purchased from Biotool (Houston, TX, USA). TransZol Up (RNAiso Plus) was purchased from TransGen Biotech Co., Ltd. (Beijing, China). The inhibitors SP600125 and LY294002 were purchased from SelleckChem (Houston, TX, USA).

### Experimental animals

40 specific pathogen-free (SPF) female Sprague Dawley (SD) rats aged 3.5 months and weighing 220–220 g were provided by the Experimental Animal Center of Dalian Medical University (Certificate of Conformity: No. SCXK 2008-0002) (Dalian, China). All experiments were approved and reviewed by the Animal Care and Use Committee of Dalian Medical University, and all experimental procedures were performed strictly in accordance with Legislation Regarding the Use and Care of Laboratory Animals of China. The rats were housed individually and maintained under environmentally controlled conditions: 12-hour light/dark cycle, temperature range of 22–25 °C, and humidity range of 45–55%. The animals had unrestricted access to tap water and food. After seven days of acclimatization, the rats were randomly divided into five groups (n = 8 per group): group A (SHAM group), group B (DEX 2.5 mg/kg group), group C (DEX 2.5 mg/kg + LA 200 mg/kg), group D (DEX 2.5 mg/kg + LA 100 mg/kg), group E (DEX 2.5 mg/kg + ALN 1 mg/kg). In group A, the rats received intramuscular injections of normal saline on their vastus twice a week. The next four groups received intramuscular injections of dexamethasone sodium phosphate on their vastus twice a week. Group A and group B were given 0.5% CMC-Na per day by gavage, and group C and group D were given LA (dissolved with 0.5% CMC-Na) per day by gavage. Group E was given ALN (dissolved with 0.5% CMC-Na) per day by gavage. All the treatments continued for nine weeks. At the end of treatment, the rats were sacrificed, and their 4–6 lumbar vertebrae, femur, tibia, and serum were collected for analysis.

### Bone biomechanical evaluation

The three-point bending test was done using a computer-controlled mechanical testing machine (SANS-10404043, Shenzhen, China) equipped with software (SANS-PowerTestDooc) to evaluate the bone mechanical strength on the femur. The conditions were as follows: sample space = 18 mm and plunger speed = 2.0 mm/min at 22–25 °C. The curves of load-displacement were plotted, and the maximum load was calculated based on this curve. The parameters were obtained through a computer-assisted analyser installed in the load cell.

### Micro-CT imaging

The trabecular micro-architectural properties of the femurs were scanned using *ex vivo* micro-CT tomography (Micro-CT, ZKKS-MCT SHARP, Zhongkekaisheng Medical Technology Co., Ltd., Guangzhou, China). The condition of the scan was set at 60 Kv X-ray voltage and 30 W X-ray power. An aluminium filter of 0.5 mm and a 2.97-s exposure time were used. The images and CT data were analysed by Medproject software. The parameters of the trabecular bone, including tissue mineral content (TMC), trabecular bone mineral content (Tb.BMC.), bone volume fraction (BV/TV), trabecular number (Tb.N.), trabecular thickness (Tb.Th.), trabecular separation (Tb.Sp.) and trabecular pattern factor (Tb.pf.), were assessed in the region of interest (ROI). A reference slice of growth plate (GP) was selected as the ROI. The ROI was set below the GP level, and related setting parameters included the number of slices (20), length (3.5 mm), width (2.2 mm), thickness (0.6 mm) and the total volume (4.84 mm^3^). The procedures were implemented using a method previously described^20, 52^.

### Histomorphometric analysis

The right femurs and right tibias were obtained and fixed using formalin (10%) for 72 h. Then, they were decalcified using EDTA (10%) for 4 weeks and embedded in paraffin. They were then cut into serial 5-μm thick longitudinal sections. Following H&E staining, the sections were observed with an inverted microscope (Olympus, Tokyo, Japan), and the images were captured using TSview software (Tucsen, Fuzhou, China).

### Measurements of BMD and BMC

The bone mineral content (BMC) and bone mineral density (BMD) of the femurs, tibia and lumbar vertebrae were measured. The BMC and BMD were measured using a dual energy X-ray absorption meter (DEXA) (Hologic, Bedford, MA) and obtained using a small animal protocol software program. The BMD was calculated based on the BMC.

### Biochemical markers of bone formation

To evaluate bone formation using the serum, osteocalcin (OCN) was measured using a rat OCN enzyme-linked immunosorbent assay (ELISA) kit (Westang Bio-Tech Co., Ltd, Shanghai, China). The manufacturer’s protocol was followed.

### Cell culture

Pre-osteoblast cells (MC3T3-E1) were purchased from the Institute of Biochemistry and Cell Biology (CAS, Shanghai, China). MC3T3-E1 cells were maintained in DMEM supplemented with FBS (10%), penicillin (100 U/ml) and streptomycin (100 U/ml). The cells were cultured at 37 °C in a humidified atmosphere of CO_2_ (5%). This medium was changed every 2 to 3 days. Culture medium containing beta-glycerophosphate (10 mM) and ascorbic acid (50 mg/L) was used to initiate differentiation and mineralization.

### ALP activity assay

MC3T3-E1 cells were seeded in 24-well culture plates and were treated with LA (0.4 mM, 0.2 mM and 0.1 mM) or ALN (10 nM) while being treated with DEX (10 μM). After 7 days, the cells were gently washed with ice-cold PBS twice. The cells were lysed with 0.2% Triton X-100. After centrifugation at 14,000 × g for 10 min at 4 °C, the ALP activity in the supernatant was detected using an ALP activity assay kit, and the total protein concentration was measured using a BCA-protein assay kit.

### Mineralization assay

The mineralization nodules were measured using Alizarin Red Staining. MC3T3-E1 cells were seeded in 6-well culture plates (1 × 10^6^ cells/well) and were treated with LA (0.4 mM, 0.2 mM and 0.1 mM) or ALN (10 nM) while being treated with DEX (10 μM). After 21 days, the culture plate was gently washed with ice-cold PBS twice, fixed with ice-cold ethanol (100%) at −20 °C for one hour, and washed three times with distilled water. Alizarin Red S dye (0.1%, pH 4.2) was added for ten hours at room temperature. Lastly, the culture plate was washed with distilled water, and the nodules were photographed under an inverted microscope (Olympus, Tokyo, Japan) using TSview software (Tucsen, Fuzhou, China). Five fields of view were chosen randomly to determine the mineral capacity for each group.

### Measurement of ROS generation

MC3T3-E1 cells were treated with LA (0.4 mM, 0.2 mM and 0.1 mM) or ALN (10 nM) for 24 h while being treated with DEX (10 μM) for 24 h. After incubation, the level of intracellular ROS was tested using the fluorescent probe 2,7-dichlorofluorescein diacetate (DCFH-DA). The relative levels of fluorescence were measured using a FACS Calibur Flow Cytometer (Becton, Dickinson and Company, Franklin Lakes, NJ).

### Measurement of MDA, SOD, GSH and LDH

The cellular MDA content, SOD activity, and GSH content; the culture solution supernatant LDH activity; and the serum MDA content, SOD activity, GSH content, and LDH activity were measured using corresponding assay kits (Nanjing Jiancheng Bioengineering Institute) according to the manufacturer’s protocols.

### Western blot analysis

Whole proteins and phosphorylated proteins from the femur or MC3T3-E1 cells were extracted according to the protocol of the corresponding assay kits (KeyGen Biotech, Nanjing, China). 10–30 µg of protein extracts were separated by SDS-PAGE gels (10%, 12%, 15%) and transferred onto polyvinyl difluoride (PVDF) membranes. After transfer, the membranes were blocked in non-fat milk (5%) in Tris-buffered saline (TBS)/Tween-20 (0.2%)(T-TBS) for 2 hours at 37 °C. Then, the membrane was immunoblotted with a primary antibody overnight at 4 °C. Following washing, the membrane was incubated with an anti-rabbit antibody for 2 hours at room temperature. The proteins were detected using an ECL Western Blotting Detection kit. The relative expression levels were normalized to the level of GAPDH protein as an internal control gene. The membranes were cut to detect the control proteins.

### qRT-PCR measurements of mRNA expression

The total RNA of the femur or MC3T3-E1 cells was isolated using TransZol Up (RNAiso Plus). 1 μl of total RNA from each sample, 5 × qRT SuperMix and RNase-free water were mixed gently. The sample was then incubated at 42 °C for 15 min and then incubated at 85 °Cfor 2 min. Then, the sample was chilled on ice until it reached room temperature. 2 μl of cDNA template, specific primers (5 μM) and distilled water (dH_2_O) were mixed gently. Then, qRT-PCR was performed using an ABI Prism 7500 (Applied Biosystems, Foster City, CA). The following cycle parameters were used: hold time of 95 °C for 5 mins and 45 cycle of 95 °Cfor 15 s and 60 °C for 30 s. The sequences for all target gene primers were purchased commercially. GAPDH was used as an internal control (TaKaRa, Dalian, China). The primers used are shown in Table 1 and Table 2. The 2^−ΔΔCT^ method was used to analyse the gene expression levels relative to GAPDH.

**Table 1:** Primers for qRT-PCR in vivo.

Gene	Primer sequence (5′-3′)
OPG	Forward: gaagatcagcccagacgagatt
	Reverse: tgctcgctgggtttgca
RANKL	Forward: agcgcttctcaggagttcca
	Reverse: gccgggccacatcga
COL-I	Forward: gcctcccagaacatcaccta
	Reverse: gcagggacttcttgaggttg
OCN	Forward: caagcaggagggcaataaggg
	Reverse: cgtcacaagcagggttaagc
BMP-2	Forward: tgaacacagctggtctcagg
	Reverse: accccacatcactgaagtcc
Runx2	Forward:ccaggcatttcatccctcact
	Reverse:gtagggtggtggcaggtacgt
OSX	Forward: aaggcagttggcaatagtgg
	Reverse: tgaatgggcttcttcctcag
LRP5	Forward: gacatttactggcccaatgg
	Reverse: ctgccctccaccaccttct
Beta-catenin	Forward: ggaaagcaagctcatcattct
	Reverse: agtgcctgcatcccacca
GAPDH	Forward: tatgactctacccacggcaa
	Reverse: atactcagcaccagcatcacc

**Table 2:** Primers for qRT-PCR *in vitro*.

Gene	Primer sequence (5′-3′)
OPG	Forward: ttacctggagatcgaattctgcttg
	Reverse: gtgctttcgatgaagtctcagctg
RANKL	Forward: gcagcatcgctctgttcctgta
	Reverse: cctgcaggagtcaggtagtgtgtc
COL-I	Forward: gcatggccaagaagacatcc
	Reverse: cctcgggtttccacgtctc
OCN	Forward: ctggctgcgctctgtctct
	Reverse: tgcttggacatgaaggctttg
BMP-2	Forward: tgaggattagcaggtctttg
	Reverse: cacaaccatgtcctgataat
Runx2	Forward:tcttcacaaatcctccccaagt
	Reverse:gaatgcgccctaaatcactga
OSX	Forward: tggccatgctgactgcagcc
	Reverse: tgggtaggcgtcccccatgg
LRP5	Forward: ctgccaggatcgctctgatg
	Reverse: acactgttgcttgatgaggacacac
Beta-catenin	Forward: gccacaggattacaagaagc
	Reverse: ccaccagagtgaaaagaacg
GAPDH	Forward: gaccacagtccatgccatcac
	Reverse: gctgttgaagtcgcaggagac

### Statistical analysis

The results are expressed as the mean ± SD. All statistical analyses were performed using SPSS 13.0 (Statistical Package for the Social Sciences, Chicago, IL, USA). All the data were compared in multi-groups using a one-way ANOVA, followed by the LSD (least significant difference) post-test. Differences were considered to be statistically significant at *P* < 0.05.

## Electronic supplementary material

Supplement information
