# Supplementary material for: The osteogenesis-promoting effects of alpha-lipoic acid against glucocorticoid-induced osteoporosis through the NOX4, NF-kappaB, JNK and PI3K/AKT pathways
Source: Sci Rep. 2017 Jun 13;7:3331. doi: 10.1038/s41598-017-03187-w (PMC5469800; doi:10.1038/s41598-017-03187-w)
Supplement: Supplementary file 1 — Supplement information [file 41598_2017_3187_MOESM1_ESM.doc]

# The osteogenesis-promoting effects of alpha-lipoic acid against glucocorticoid-induced osteoporosis through the NOX4, NF-kappaB, JNK and PI3K/AKT pathways

#

Shi-Yu Lu^1^, Chang-Yuan Wang^1^, Yue Jin^1^, Qiang Meng^1^, Qi Liu^1^, Zhi-hao Liu^1^, Ke-Xin Liu^1^, Hui-Jun Sun^1^*, and Mo-Zhen Liu^2^**

^1^Department of Clinical Pharmacology, College of Pharmacy, Dalian Medical University, Dalian,China

^2^Department of Orthopaedics, First Affiliated Hospital, Dalian Medical University, Dalian, China

*Correspondence to: Hui-Jun Sun, Department of Clinical Pharmacology, College of Pharmacy, Dalian Medical University, 9 West Section, Lvshun South Road, Lvshunkou District, Dalian 116044, China.

Tel/fax: +86 411 86110413.

E-mail: sunhuijun@dlmedu.edu.cn

**Correspondence to: Mo-Zhen Liu, Department of Orthopaedics, First Affiliated Hospital, Dalian Medical University, Zhongshan Road No. 222, Dalian 116011, China.

Tel/fax: +86 411 83635963.

E-mail: mozhenliu@hotmail.com

**Supplementary information**

**
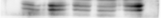
**

**Fig S1. Caspase-3 in fig 2E of the manuscript.**

**
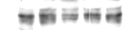
**

**Fig S2. Caspase-9 in fig 2F of the manuscript.**

**
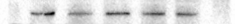
**

**Fig S3. Bcl-2 in fig 2G of the manuscript.**

**
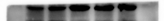

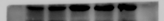

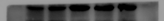
**

**Fig S4. GAPDH in fig 2E-G of the manuscript.**

**
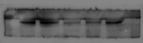
**

**Fig S5. Nox4 in fig 5A of the manuscript.**

**
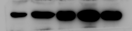

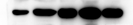
**

**Fig S6. GAPDH in fig 5A of the manuscript.**

**
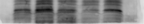
**

**Fig S7. P-P65 in fig 5B of the manuscript.**

**
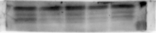
**

**Fig S8. T-P65 in fig 5B of the manuscript.**

**
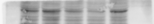
**

**Fig S9. P-JNK in fig 5C of the manuscript.**

**
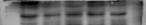
**

**Fig S10. T-JNK in fig 5C of the manuscript.**

**
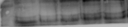
**

**Fig S11. P-AKT in fig 5D of the manuscript.**

**
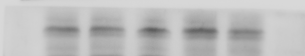

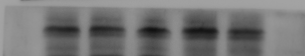
**

**Fig S12. T-AKT in fig 5D of the manuscript.**

**
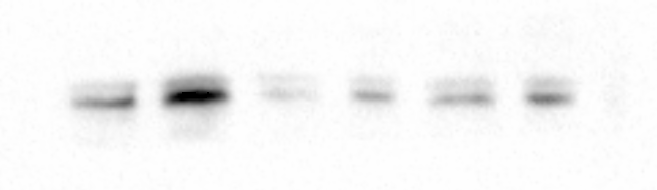
**

**Fig S13. Caspase-3 in fig 6F of the manuscript.**

**
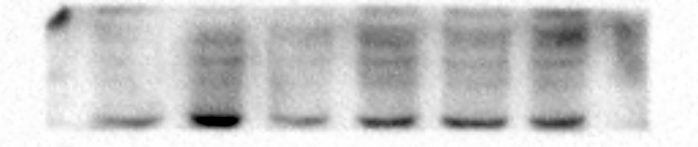
**

**Fig S14. Caspase-9 in fig 6G of the manuscript.**

**
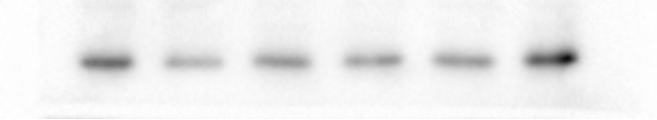
**

**Fig S15. Bcl-2 in fig 6H of the manuscript.**

**
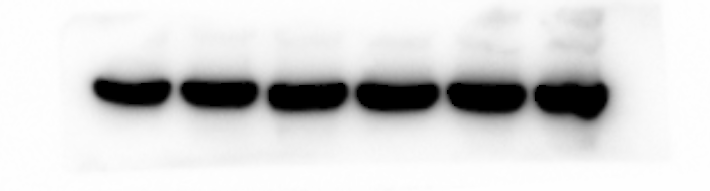

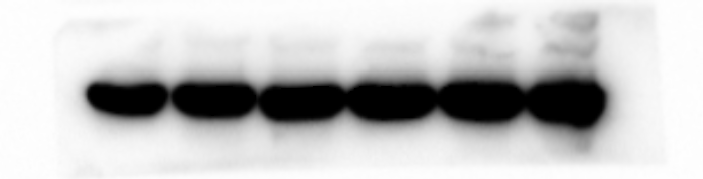

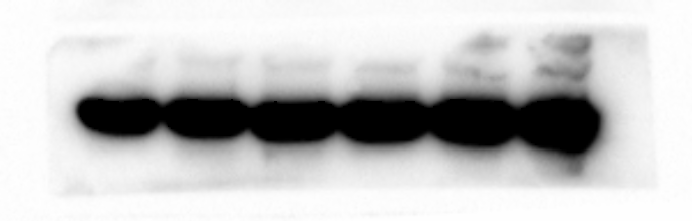
**

**Fig S16. GAPDH in fig 6F-H of the manuscript.**

**
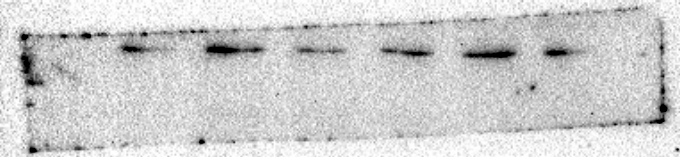
**

**Fig S17. Nox4 in fig 9A of the manuscript.**

**
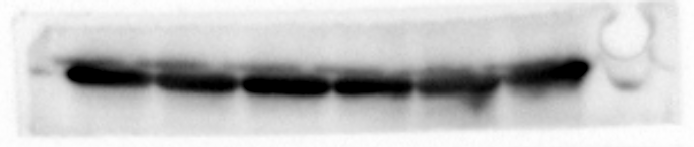
**

**Fig S18. GAPDH in fig 9A of the manuscript.**

**
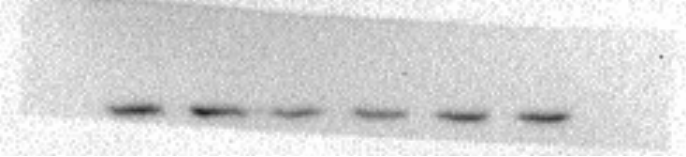
**

**Fig S19. P-P65 in fig 9B of the manuscript.**

**
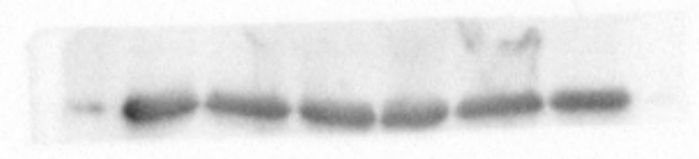
**

**Fig S20. T-P65 in fig 9B of the manuscript.**

**
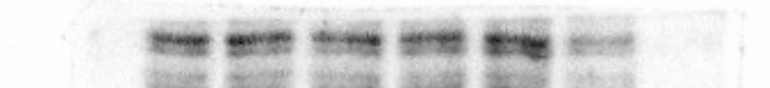
**

**Fig S21. P-JNK in fig 9C of the manuscript.**

**
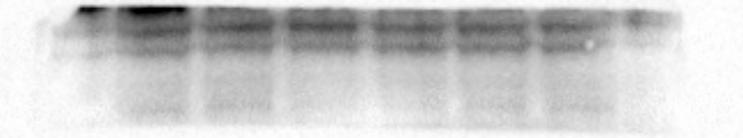
**

**Fig S22. T-JNK in fig 9C of the manuscript.**

**
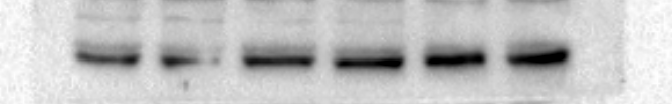
**

**Fig S23. P-AKT in fig 9D of the manuscript.**

**
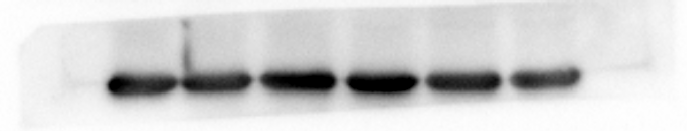

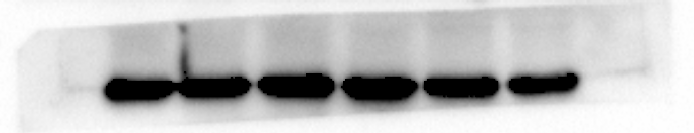
**

**Fig S24.T-AKT in fig 9D of the manuscript.**
